# Clinical features and genetic spectrum in Chinese patients with recessive hereditary spastic paraplegia

**DOI:** 10.1186/s40035-019-0157-9

**Published:** 2019-06-26

**Authors:** Qiao Wei, Hai-Lin Dong, Li-Ying Pan, Cong-Xin Chen, Yang-Tian Yan, Rou-Min Wang, Hong-Fu Li, Zhi-Jun Liu, Qing-Qing Tao, Zhi-Ying Wu

**Affiliations:** 1 Department of Neurology and Research Center of Neurology in Second Affiliated Hospital, and Key Laboratory of Medical Neurobiology of Zhejiang Province, Zhejiang University School of Medicine, 88 Jiefang Road, Hangzhou, 310009 China; 2 Longyan First Hospital, Fujian Medical University, Longyan, China; 3 Department of Neurology and Institute of Neurology, Huashan Hospital, Shanghai Medical College, Fudan University, Shanghai, China; 4 Joint Institute for Genetics and Genome Medicine between Zhejiang University and University of Toronto, Zhejiang University, Hangzhou, China

**Keywords:** Hereditary spastic paraplegia, Autosomal recessive, Targeted next-generation sequencing, Chinese, Genetic spectrum, Phenotype

## Abstract

**Background:**

Although many causative genes of hereditary spastic paraplegia (HSP) have been uncovered in recent years, there are still approximately 50% of HSP patients without genetically diagnosis, especially in autosomal recessive (AR) HSP patients. Rare studies have been performed to determine the genetic spectrum and clinical profiles of recessive HSP patients in the Chinese population.

**Methods:**

In this study, we investigated 24 Chinese index AR/sporadic patients by targeted next-generation sequencing (NGS), Sanger sequencing and multiplex ligation-dependent probe amplification (MLPA). Further functional studies were performed to identify pathogenicity of those uncertain significance variants.

**Results:**

We identified 11 mutations in HSP related genes including 7 novel mutations, including two (p.V1979_L1980delinsX, p.F2343 fs) in *SPG11*, two (p.T55 M, p.S308 T) in *AP5Z1*, one (p.S242 N) in *ALDH18A1,* one (p.D597fs) in *GBA2*, and one (p.Q486X) in *ATP13A2* in 8 index patients and their family members. Mutations in *ALDH18A1, AP5Z1, CAPN1* and *ATP13A2* genes were firstly reported in the Chinese population. Furthermore, the clinical phenotypes of the patients carrying mutations were described in detail. The mutation (p.S242 N) in *ALDH18A1* decreased enzyme activity of P5CS and mutations (p.T55 M, p.S308 T) in *AP5Z1* induced lysosomal dysfunction.

**Conclusion:**

Our results expanded the genetic spectrum and clinical profiles of AR-HSP patients and further demonstrated the efficiency and reliability of targeted NGS diagnosing suspected HSP patients.

**Electronic supplementary material:**

The online version of this article (10.1186/s40035-019-0157-9) contains supplementary material, which is available to authorized users.

## Background

Hereditary spastic paraplegia (HSP) is a rare inherited neurological disorder with heterogeneous clinical phenotypes which are classified into pure and complex forms [1]. Pure HSP is characterized by a pyramidal syndrome with progressive paraparesis and spasticity of the lower limbs. Patients with complex HSP present extremity spasticity with various symptoms such as seizures, deafness, cerebellar dysfunction, cognitive retardation, cataract and peripheral neuropathy [2–5]. To date, more than 70 different genetic types of HSP have been identified, and all modes of inheritance including autosomal dominant (AD), autosomal recessive (AR), X-linked or non-Mendelian mitochondrial maternal transmission have been observed in HSP patients [6–8]. Clinical symptoms in cases of AD-HSP mainly present pure type, and about 40–50% of which are caused by mutations in the *SPAST* gene (*SPG4*), while the AR-HSP is often associated with complex type that is usually caused by mutations in the *SPG11* gene [9, 10]. Although many causative genes have been uncovered in the recent years, there are still approximately 50% of HSP patients without genetically diagnosis, especially in AR/sporadic HSP patients [11]. Rare studies have been performed to determine the genetic spectrum of AR/sporadic HSP patients in the Chinese population [12–14]. In addition, phenotypes of AR/sporadic HSP patients with rare causative genes have seldom been reported in the Chinese population.

In our previous study, we performed mutation screening in 55 Chinese HSP pedigrees, including 39 AD, 8 AR and 8 sporadic families, by targeted next-generation sequencing (NGS) and multiplex ligation-dependent probe amplification (MLPA). Thirty-four families including 30 AD, two AR (one with *SPG11* mutations and the other with *CYP7B1* mutations) and two sporadic (one with *SPG11* mutations, actually AR-HSP family) families were genetically diagnosed [15]. Here, we applied targeted NGS combined with Sanger sequencing and MLPA for the genetic analysis of 24 unrelated AR/sporadic HSP patients. In addition, phenotypes of AR/sporadic HSP patients with mutations in rare causative genes were described. Our results depict the genetic spectrum and clinical phenotype of AR/sporadic HSP patients in the Chinese population.

## Materials and methods

### Subjects

Twenty-four unrelated patients with clinically suspected HSP including 4 out of 6 AR-HSP and 4 out of 6 sporadic patients without pathogenic mutations reported previously [15] and 24 family members were consecutively collected in Huashan Hospital of Fudan University and Second Affiliated Hospital of Zhejiang University School of Medicine between March 2009 and May 2018. This study was approved by the Ethics Committees of these two hospitals. Written informed consents were obtained from all patients. Patients were diagnosed with HSP based on Harding’s criteria [3] and evaluated by at least two senior neurologists. Subjects with autosomal dominant family history or pure sporadic HSP were excluded. Age at onset (AAO) was based on the first symptom, such as leg numbness, gait abnormality or walk disabled. Most of probands underwent biochemical tests, electromyogram (EMG) and standard 1.5 T/3 T magnetic resonance imaging (MRI). In addition, 2500 Chinese healthy controls (49% males and 51% females) were involved.

### Targeted next-generation sequencing and sanger sequencing

Genomic DNA was extracted from a peripheral EDTA-treated blood sample of each patient by using QIAamp blood genomic extraction kits (Qiagen, Hilden, Germany). A customized panel (Roche, Madison, USA) of genes related to HSP was designed (Additional file 1: Table S1). Deep sequencing was executed on the Illumuna HiSeq2000 platform (Genergy Biotechnology, Shanghai, China). The annotations and analyses of sequenced reads were carried out as described in our previous publications [16, 17]. After annotation, data on the detected variants were extracted from publicly available databases including the 1000 Genomes Project, the Exome Aggregation Consortium (ExAC) Browser, and the Single Nucleotide Polymorphism (dbSNP) Database. To predict the possible protein functional changes, three software programs (SIFT, PolyPhen2 and Mutation Taster) were used. Probable variants were confirmed through Sanger sequencing on ABI 3500XL DX DNA sequences. Variants for co-segregation analysis were confirmed through all available familial members.

### Multiplex ligation-dependent probe amplification assay

Patients remaining negative following targeted NGS were further examined for large deletions or duplications of *SPAST*, *ATL1*, *REEP1*, *PGN* and *SPG11* through the MLPA analysis, using commercially available MLPA kits (SALSA P165-C2; SALSA P213-B2; SALSA P306-B1; MRC-Holland, the Netherlands) according to the manufacturer’s recommendations.

### Plasmid constructs

The coding sequence of the human wild-type (WT) *ALDH18A1* gene (NM_002860) and *AP5Z1* gene (NM_014855) were cloned into pcDNA3.1/myc-his vector, respectively. The variants within *ALDH18A1* (p.S242 N and p.V243 L) and *AP5Z1* (p.T55 M and p.S308 T) were introduced to plasmids using PCR mutagenesis.

### Cell culture and transfection

Hela cells and HEK293 cells were cultured at 37 °C under 5% CO_2_ in DMEM (HyClone) supplemented with 10% fetal bovine serum (Gibco) and transiently transfected with WT and mutant plasmids using Lipofectamine 3000 reagent (Invitrogen) according to the manufacture’s protocol. Forty-eight hours after transfection, transfected cells were collected for further analysis.

### Immunofluorescence analysis and quantification

Hela cells respectively transfected with WT *ALDH18A1* plasmid and mutant *ALDH18A1* plasmids were cultured in glass-bottomed dishes and mitochondria was visualized with MitoTracker red probe (Invitrogen). Then culture medium was removed from the transfected Hela cells and the cells were washed three times with ice-cold phosphate-buffered salin (PBS). The cells were fixed with 4% paraformaldehyde for 20 min, permeabilized with 0.1% Triton X-100, blocked with 5% donkey serum for 1 h, and immunostained with anti-His (1:1000) (Abmart) and secondary anti-mouse IgG Alexa Fluor488 antibody (1:500) (Life Technologies). Hela cells respectively transfected with WT *AP5Z1* plasmid and mutant *AP5Z*1 plasmids were immunostained with anti-LAMP1 (1:1000) (Abcam) and secondary anti-mouse IgG Alexa Fluor488 antibody (1:500) (Life Technologies). Fluorescence images were captured by Olympus FV3000 OSR confocal system. More than 100 cells per visual field were quantified for each condition using Image-J software (NIH). Quantification of the particle number, fluorescence intensity and spot area was performed by a person blind to the experiment. Experiments were replicated three times.

### Transmission electron microscope

Hela cells respectively transfected with WT *AP5Z1* plasmid and mutant *AP5Z*1 plasmids were grown on plastic dishes and harvested by trypsinization. All samples were fixed in 2.5% glutaraldehyde for 4 h, washes for 10 min three times with PBS, post-fixed in 1% osmium solution, and embedded in epoxy resin overnight. Images were obtained using H7500 transmission electron microscope (TEM) (Hitachi).

### Western blot analysis

Protein samples from HEK293 cells were resolved by 10% SDS-polyacrylamide gel electrophoresis (SDS-PAGE), transferred to Polyvinylidene fluoride (PVDF) membranes and blotted with the desired antibodies. Specific bands were detected with anti-His (1:1000) (Abmart), anti-GAPDH (1:5000) (Abmart), respectively. Quantification of density in each band was performed as detailed previously [18].

### Biochemical assay

The blood samples from the patient carrying *ALH18A1* mutation and four gender matched healthy controls were centrifuged (3000 rpm; 5 min), then serum was fractionated and stored at − 80 °C. Activity of delta-1-pyrroline-5-carboxylate synthase (P5CS), an enzyme encoded by *ALDH18A1*, was measured by using a human enzyme linked immunosorbent assay (ELISA) kit (Shanghai Jianglai Biotech).

### Statistical analysis

Data are presented as mean ± standard error. One-way ANOVA with Dunnett’s multiple comparisons test was used when the interaction had significant differences. *P* values of < 0.05 were regarded as significant. The statistical analysis was performed using the Prism software (Graphpad prism for Mac).

## Results

### Clinical manifestations of patients

Twenty-four unrelated probands were recruited in our study and 87.5% (21/24) of them came from southeast of China. The personal and medical histories of all patients were summarized in Additional file 2: Table S2. Among these patients, 7 out of 24 (29%) patients presented with pure HSP while 17 out of 24 (71%) presented with complex HSP. The symptoms found in complex HSP patients include neuropathy (7/17), cerebellar signs (6/17), mental retardation (5/17), tremor (3/17), and visual impairment (1/17). The mean AAO was 20 years (range 1–58 years) and average duration was 11 years (range 1–45). Nineteen were sporadic cases and five had a family history compatible with AR inheritance, indicating that at least two affected individuals were in a single generation. Nine out of 24 patients were born in consanguinity families.

### Identification of mutations

**Fig. 1:**
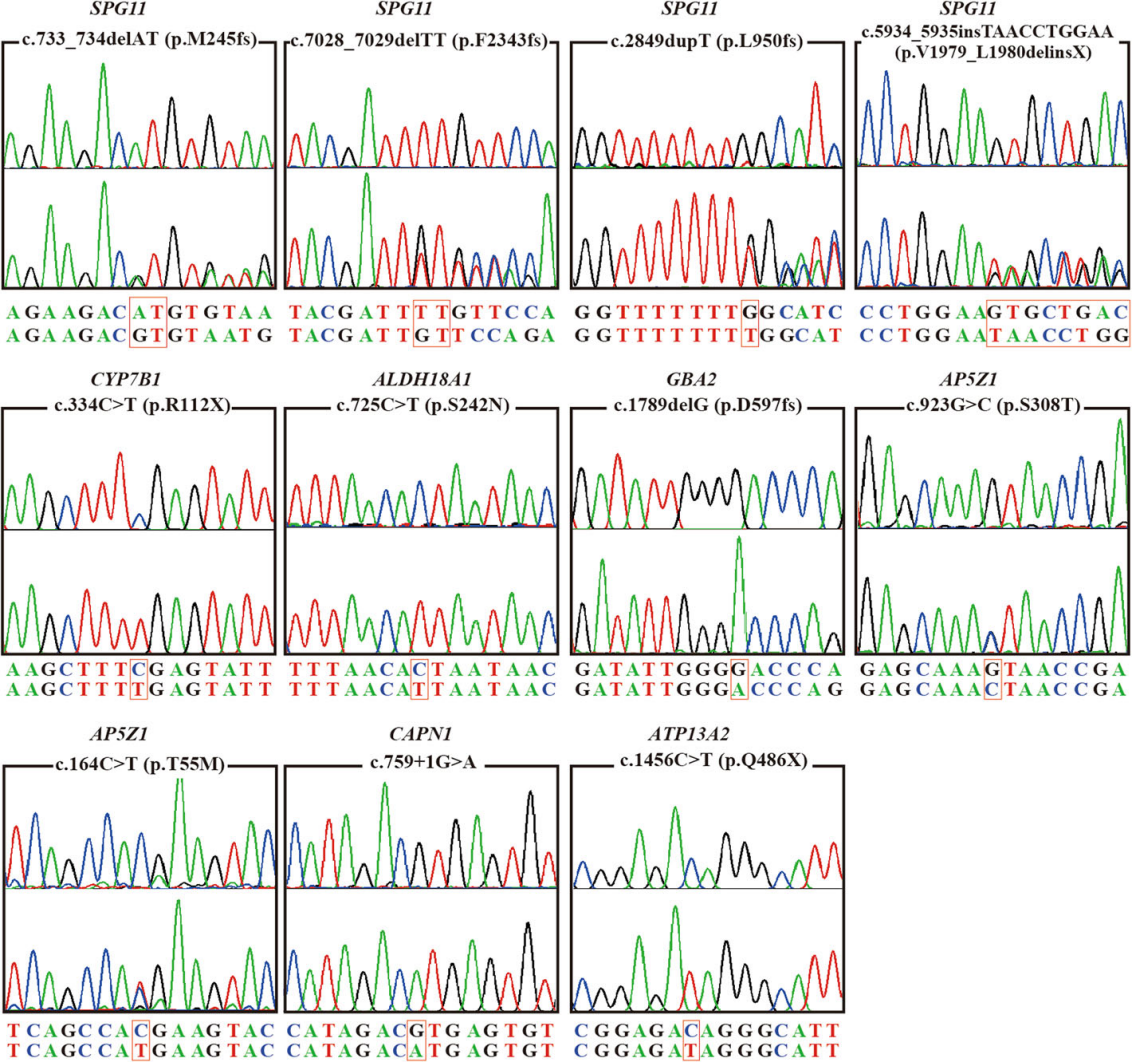
Chromatograms of 11 mutations identified in the present study. The upper chromatogram in each frame represents the reference sequence, and the lower one depicts the mutant sequence. The p.R112X in *CYP7B1*, p.S242 N in *ALDH18A1*, p.D597fs in *GBA2,* c.759 + 1G > A in *CAPN1*, and p.Q486X*A* in *TP13A2* are homozygous

Targeted NGS was applied in this study. The coverage of the fraction of target bases indicated that 87.70% target bases had >50x coverage. The mean coverage of target bases ranged from 73.74 to 264.54. Eleven variants (Table 1) in seven known genes related to HSP were identified in eight index patients (Table 2). Seven variants were novel and four (p.M245 fs, p.L950 fs in *SPG11*, p.R112X in *CYP7B1*, c.759 + 1G > A in *CAPN1*) were previously reported [19–22]. According to the American College of Medical Genetics and Genomics (ACMG) standards [23], two *SPG11* variants (p.V1979_L1980delinsX and p.F2343 fs), one *GBA2* variant (p.D597fs), and one *ATP13A2* variant (p.Q486X) were classified as pathogenic mutations, whereas two *AP5Z1* variants (p.T55 M and p.S308 T) and one *ALDH18A1* variant (p.S242 N) were classified as uncertain significance variants. Four known variants including two *SPG11* variant (p.M245 fs, p.L950 fs), one *CYP7B1* variant (p.R112X) and one *CAPN1* variant (c.759 + 1G > A) were identified as pathogenic mutations (Fig. 1). Therefore, all 11 variants found in this study were disease causative mutations.

**Table 1:** Features of variants identified in this study

Gene	dbSNP ID	Exon	Nucleotide change	Amino acid change	Mutation type	Predicted Impact	1000 Genomes	ExAc	GnomAD	Family Segregation	Final Classification^b^
*SPG11*	rs312262720	4	c.733_734delAT^a^	p.M245 fs^a^	Deletion	NA/NA/NA	absent	0.0001	6.133E-5	yes	Pathogenic
*SPG11*	rs312262751	16	c.2849dupT^a^	p.L950 fs^a^	Insertion	NA/NA/NA	absent	absent	4.07e-6	yes	Pathogenic
*SPG11*	rs749652788	31	c.5934_5935insTAACCTGGAA	p.V1979_L1980delinsX	Insertion	NA/NA/NA	absent	8.26E-6	4.069E-6	yes	Pathogenic
*SPG11*	NA	39	c.7028_7029delTT	p.F2343 fs	Deletion	NA/NA/NA	absent	absent	absent	yes	Pathogenic
*CYP7B1*	rs200737038	3	c.334C > T^a^	p.R112X^a^	Nonsense	NA/NA/A	0.0002	0.0002	0.0001422	yes	Pathogenic
*ALDH18A1*	NA	7	c.725G > A	p.S242 N	Missense	T/P/D	absent	absent	absent	NA	Pathogenic
*GBA2*	NA	11	c.1789delG	p.D597fs	Deletion	NA/NA/D	absent	absent	absent	yes	Pathogenic
*AP5Z1*	rs182694738	2	c.164C > T	p.T55 M	Missense	D/D/D	0.0004	0.0001	0.0001733	NA	Likely Pathogenic
*AP5Z1*	rs1035120004	7	c.923G > C	p.S308 T	Missense	T/D/D	absent	absent	absent	NA	Likely Pathogenic
*CAPN1*	NA	6	c.759 + 1G > A^a^	–	splicing	NA/NA/D	absent	absent	4.189e-6	yes	Pathogenic
*ATP13A2*	NA	15	c.1456C > T	p.Q486X	Nonsense	NA/NA/A	absent	absent	absent	yes	Pathogenic

The impact of non-synonymous protein-coding region variants were determined using prediction software including SIFT, PolyPhen-2 and Mutation Taste. SIFT results as Tolerated (T) or Deleterious (D). PolyPhen-2 results as Unknown (UN), Benign (B), Possibly Damaging (P) or Probably Damaging (D). Mutation Taste results as Tolerated (T), Disease causing (D) and Disease causing automatic (A). 1000G, 1000 Genomes Project; ExAC, Exome Aggregation Consortium; ^a^, Reported previously; ^b^, Variants were finally classified with the functional data according to ACMG guidelines. *NA* Not available

**Table 2:** Clinical data of HSP patients with mutations in HSP-related genes in this study

Patient	Sex	AAO	DD	Inheritance	Gene	Variants (known/novel)	Variant type	Phenotype	Neuropathy	UL/LL hyperreflexia	LL weakness	Ankle clonus	Babinski	Additional features
Case 1	F	24	3	sporadic	*SPG11*	p.M245 fs (known) p.F2343 fs (novel)	het het	C	–	+/+	+	+	+	dysarthria, mental retardation
Case 2	M	16	5	sporadic	*SPG11*	p.L950 fs (known) p.V1979_L1980delinsX (novel)	het het	C	–	−/−	–	+	–	tremor
Case 3	F	38	10	sporadic	*CYP7B1*	p.R112X (known)	hom	C	+	+/+	+	+	+	dysarthria, visual impairment
Case 4	F	12	20	sporadic	*ALDH18A1*	p.S242 N (novel)	hom	C	+	−/+	+	NA	+	depression and anxiety
Case 5	M	22	4	sporadic	*GBA2*	p.D597fs (novel)	hom	C	+	−/+	+	+	+	Hoffmann sign, mental retardation
Case 6	M	20	14	AR	*CAPN1*	c.759 + 1G > A (knwon)	hom	P	–	+/+	–	+	+	Hoffmann sign
Case 7	M	58	2	sporadic	*AP5Z1*	p.T55 M (novel) p.S308 T (novel)	het het	C	+	−/+	–	+	–	Hoffmann sign
Case 8	M	24	4	sporadic	*ATP13A2*	p.Q486X (novel)	hom	C	+	+/+	–	–	+	nystagmus,

*AAO* Age at onset, *DD* Disease duration, *F* Female, *M* Male, *AR* Autosomal recessive, *het* Heterozygosis, *hom* Homozygosis, *P* Pure, *C* Complex, *UL* Upper limbs, *LL* Lower limbs, + Present, − Absent; *NA* Not available

### Clinical features of patients carrying *SPG11* mutations

**Fig. 2:**
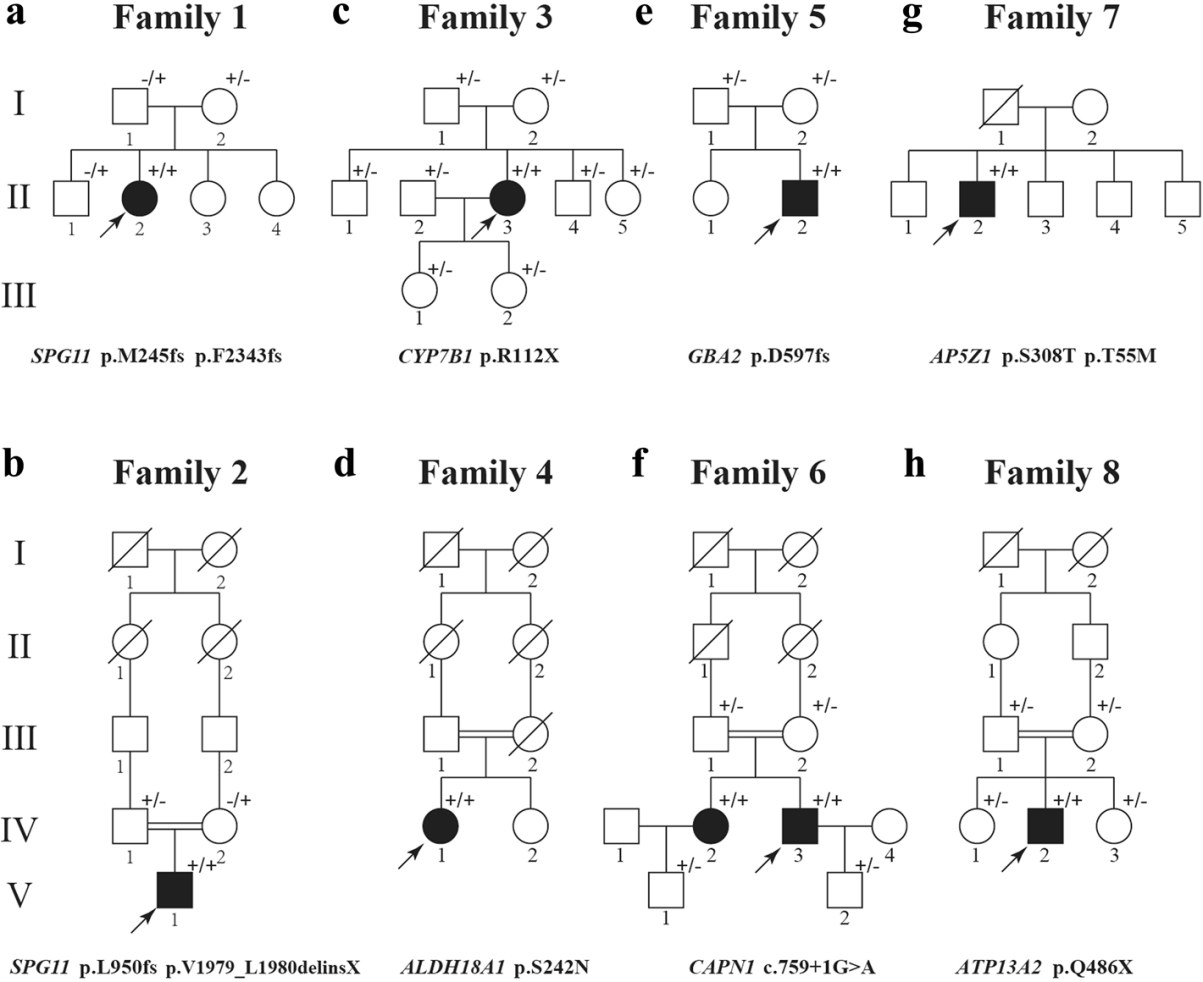
Pedigrees of 8 HSP families (**a**-**h**) in our cohort. Squares indicate males; circles indicate females; the black symbols indicate affected individuals; arrows indicate the probands. Symbol with “+/+” indicate patient. Symbol with “+/−” or “+/−” indicate mutation carrier

**Fig. 3:**
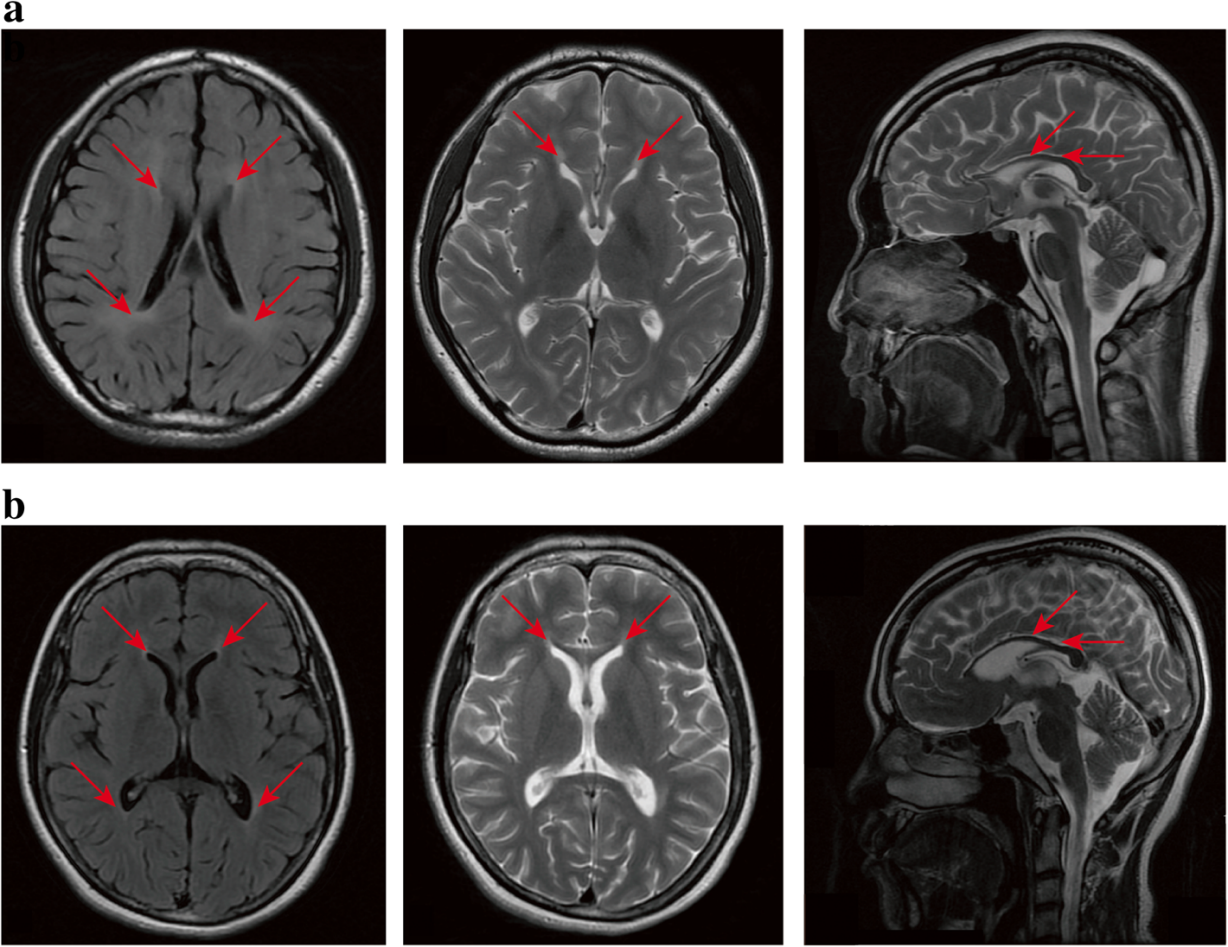
Brain MRI of case 1 and case 2. An ‘ears of the lynx’ appearance and thinning of corpus callosum were seen in case 1 (**a**) and case 2 (**b**)

Two novel pathogenic mutations and two known pathogenic mutations within *SPG11* were identified in two unrelated patients. All of them showed complex HSP phenotypes. Case 1 (II-2 in Family 1) **(**Fig. 2a**)** carrying two pathogenic mutations (p.M245 fs and p.F2343 fs) is a 26-year-old woman who showed progressive weakness of lower limbs and scissors gait two years ago. As gait abnormality progressed, she developed dysarthria, dysphagia, mental impairment, and severe cognitive impairment with mini-mental state examination (MMSE) score of 16/30. The spasticity in the lower extremities was severe while in the upper extremities was mild. She had bilateral hyperactive deep tendon reflexes and positive Hoffmann signs. Extensor plantar response and patellar clonus were observed on the left side. The brain MRI showed an ‘ears of the lynx’ appearance and thinning of corpus callosum **(**Fig. 3a**)**. Cerebellum, brainstem and cervical spinal cord were normal. Her father and elder brother carried the heterozygous p.F2343 fs mutation, while her mother carried the heterozygous p.M245 fs mutation.

Case 2 (V-1 in Family 2) **(**Fig. 2b**)** carrying two pathogenic mutations (p.L950 fs and p.V1979_L1980delinsX) is a 21-year-old man and was born in a consanguinity family. He showed normal motor and psychological milestones. It was atypical that he had tremor in the right upper limb at the age of 16. He showed progressive difficulty in walking and memory notably in his 20s. On the neurological examination, left hand tremor, scissors gait, patellar clonus and increased muscle tone were observed. Muscle strength and tendon reflexes were normal in extremities. No rigidity and bradykinesia were observed. Baclofen was prescribed at a dose of 10 mg twice a day and his muscle spasms had been effectively relieved. The brain MRI showed corpus callosum thinning and an ‘ears of the lynx’ appearance **(**Fig. 3b**)**. His father carried the heterozygous p.L950 fs mutation and his mother carried the heterozygous p.V1979_L1980delinsX mutation.

### Clinical features of patients carrying mutations within *SPG5/SPG9*/*SPG46*/ *SPG48*/*SPG76*/*SPG78* genes

Case 3 (II-3 in Family 3) **(**Fig. 2c**)** carrying a reported homozygous pathogenic mutation in *SPG5*/*CYP7B1* (p.R112X) is a 48-year-old female. Her birth history was normal with full-term delivery. At the age of 38, she began to experience stiffness in her lower limbers and gradually developed walking difficulty. Numbness and tingling were also noticed in her legs. When she was 46 years old, she presented dizziness and visual impairment. She developed dysarthria and dysphagia one year later. No muscle atrophy or autonomic symptoms were present. Physical examination revealed decreased muscle strength, increased muscle tone in the lower limbers, hyperreflexia in all limbs, and bilaterally extensor plantar responses. Hoffmann sign was observed in her right arm. Spinal MRI presented atrophy of spinal cord (T2-T10). All of her family members were carriers of the p.R112X mutation.

A novel homozygous missense mutation within *SPG9*/*ALDH18A1* (p.S242 N) was found in a 32-year-old female (Case 4, IV-1 in Family 4) **(**Fig. 2d**)**. She was a worker without exposure to any toxin. Her birth and developmental milestones were unremarkable. She could not walk fast and consistently since she was 10 years old and had an onset at the age of 20 with weakness of the lower extre mities. When she was 27 years old, she noted progressive weakness of the upper extremities. She developed agitation and irritability after pregnancy. No symptom of cutis laxa was observed. Neurological examinations revealed hypertonia and bilateral hyperactive deep tendon reflexes in the lower extremities. The EMG exhibited neuropathy. Brain MRI was normal. Her family members were not available to test.

Case 5 (II-2 in Family 5) **(**Fig. 2e**)** with a novel homozygous pathogenic *SPG46***/***GBA2* mutation (p.D597fs) is a 26-year-old man. He denied any exposure to pesticides and other toxins. He experienced walking unstable and abnormal walking posture at age of 22. Pain of lower limbs was noticed when he was 25 years old. At the same time, he started to show psychiatric disorders such as delusion of persecution, anxiety, and fear. He exhibited representative pyramidal signs upon neurological examination. Muscle strength and sensation were normal. The EMG revealed multiple peripheral nerve damage. MRI scanning showed the brain, cervical, thoracic and lumbar were normal. Both of his parents carried the heterozygous p.D597fs mutation.

One known homozygous pathogenic mutation (c.759 + 1G > A) within *SPG76*/*CAPN1* was identified in case 6 with AR-HSP (IV-3 in Family 6) **(**Fig. 2f**)** from a consanguinity family. Case 6 is a 34-year-old male patient with pure HSP. He suffered from walking problems at age of 20. As walking stiffness progressed, he showed dysarthria and dysphagia. His examinations showed a severe spastic paraplegia. He had an elder sister (IV-2 in Family 6) carrying the same homozygous *CAPN1* mutation. Her walking problem was similar to the proband. His parents carried the heterozygous mutation (c.759 + 1G > A) with no symptom.

Two novel heterozygous pathogenic mutations (p.T55 M and p.S308 T) within *SPG48*/*AP5Z1* were detected in a 60-year-old man (case 7, II-2 in Family 7) **(**Fig. 2g**)** with a two-year history of unsteady gait**.** He showed difficulty in walking stairs and running. Pyramidal syndromes of lower limbs including enhanced brisk patellar reflexes and ankle clonus were presented. Unilaterally Hoffmann sign and enhanced reflexes of upper limbs were also observed. Sensory and cerebellar signs were absent. The EMG showed neuropathy. Brain MRI revealed normal. None of his family members was available to test.

A novel homozygous mutation (p.Q486X) in SPG78/ATP13A2 was identified in case 8 (IV-2 in Family 8) **(**Fig. 2h**)**. He is a 28-year-old male and his birth and development milestones were totally normal. At the age of 24, he presented spastic quadriplegia with pain. Neurological examinations revealed gait disturbance and increased muscle tone in his lower limbs. Hyperreflexia was observed in his extremities. Nystagmus was prominent. Muscle strength and sensory were normal. His brain MRI was normal. Electrophysiological studies showed reduced nerve conduction in the peroneal nerve and the tibial nerve. His parents and sisters were normal and all carried the heterozygous mutation (p.Q486X).

### Functional analysis of *ALDH18A1* mutations

**Fig. 4:**
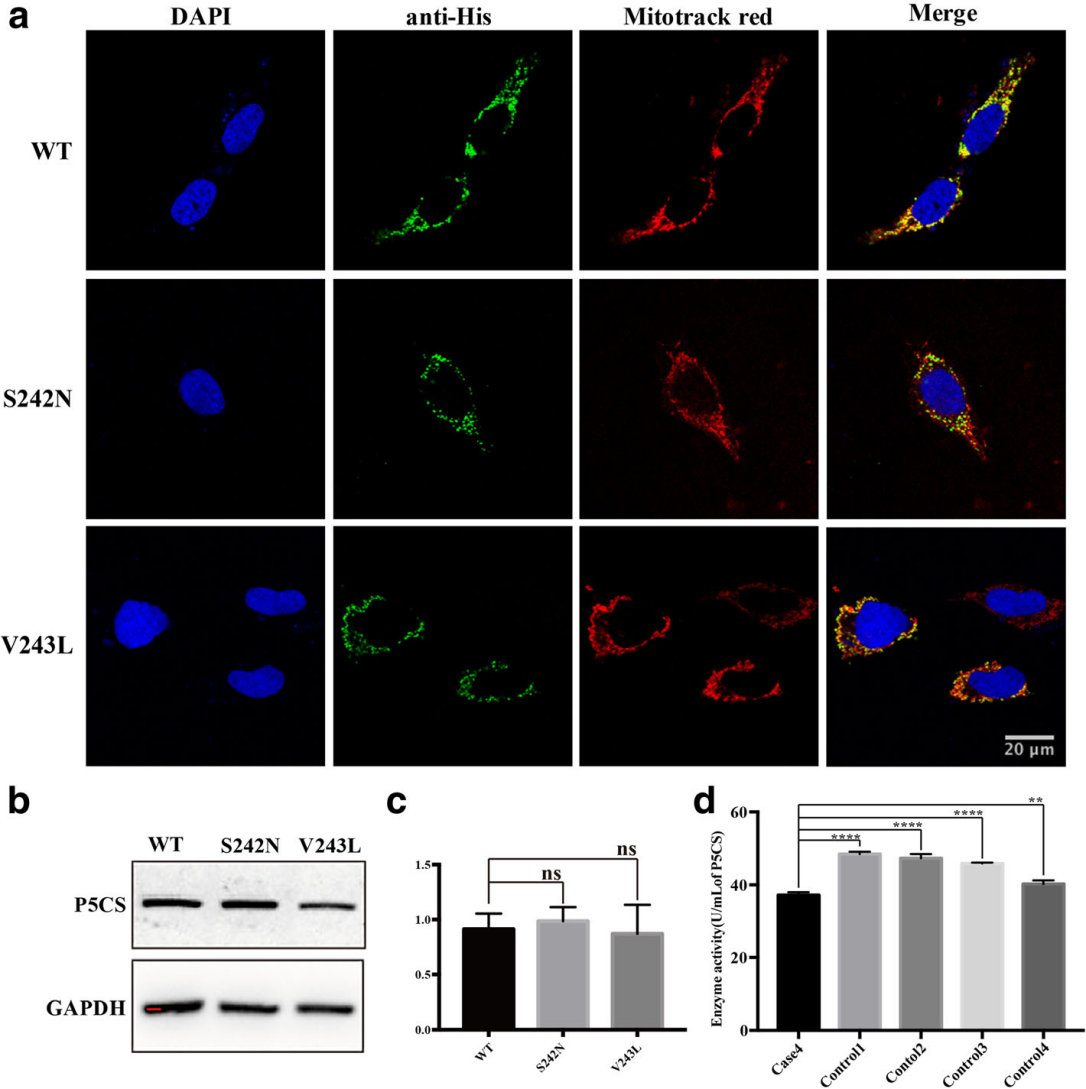
The novel homozygous variant (p.S242 N) in *ALDH18A1* decreased enzyme activity of P5CS. **a** Hela cells were respectively transfected with WT and mutant plasmids (S242 N, V243 L). Mitochondria was visualized with MitoTracker red probe and no dramatic change in mitochondrial localization was found. (**b**, **c**) HEK293 cells were respectively transfected with WT and mutant plasmids (S242 N, V243 L). No significant difference of protein level was detected between WT P5CS and mutant P5CS. **d** The serum P5CS activity of case 4 decreased as compared with that of four gender matched healthy controls. Scale bar = 20 μm. Error bars represent SEM, *p < 0.05

*ALDH18A1* encodes P5CS, an enzyme that catalyzes proline and ornithine biosynthesis. Functional analysis was performed in the novel variant (p.S242 N) within *ALDH18A1* detected in case 4. A known mutation p.V243 L was used as a positive control. After immunofluorescent staining analysis, there was no dramatic change in mitochondrial localization of mutant P5CS as compared with WT P5CS (Fig. 4a). No significant difference of protein level was detected between WT P5CS and mutant P5CS (Fig. 4b, c). However, the serum P5CS activity of case 4 decreased as compared with that of four gender matched healthy controls (*p* < 0.005) (Fig. 4d). With functional data, the p.S242 N was classified as a pathogenic variant (PS3, PM1, PM2, PP2, PP3, PP4).

### Functional analysis of *AP5Z1* mutations

**Fig. 5:**
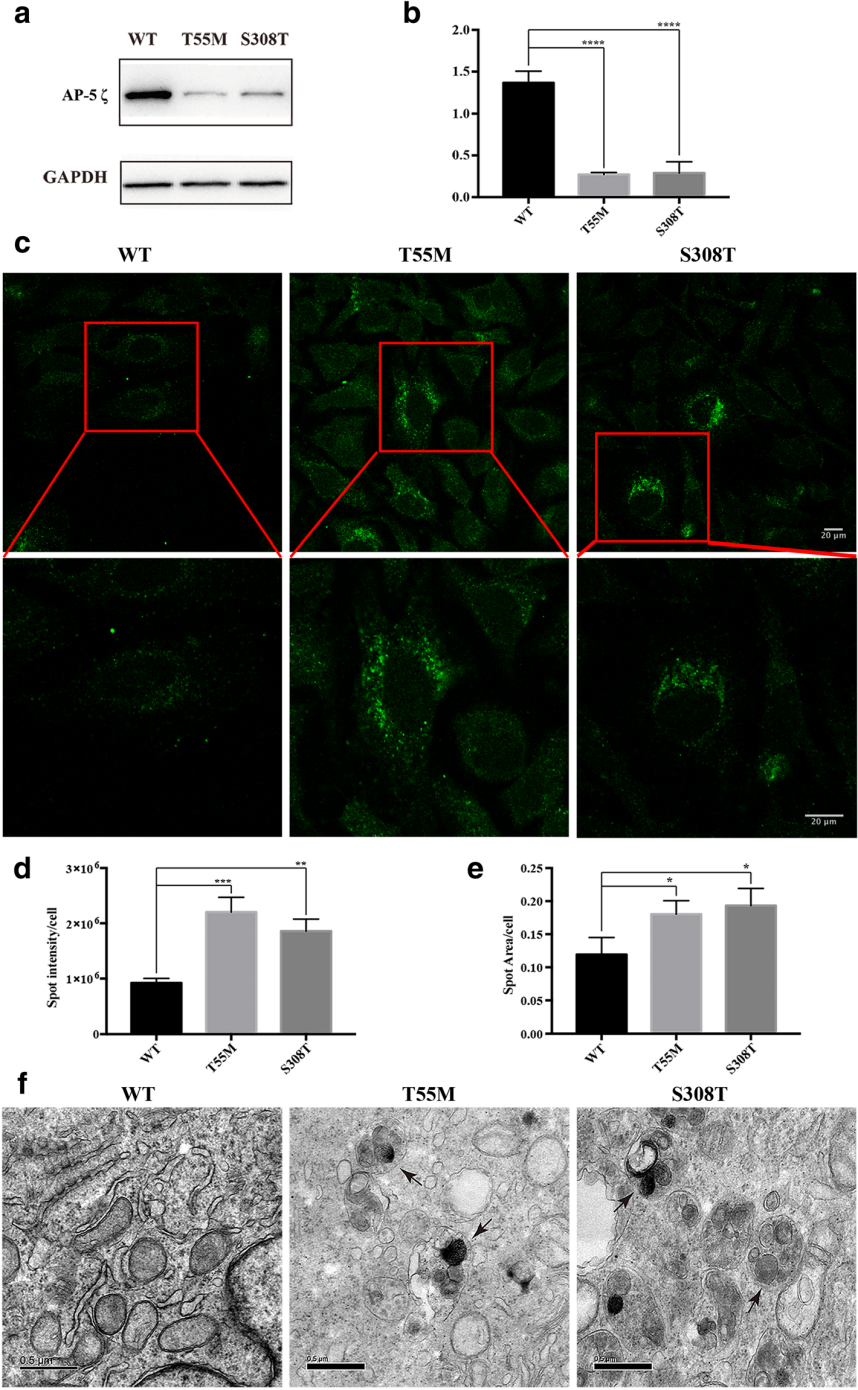
Functional analysis of both variants (p.T55 M and p.S308 T) in *AP5Z1*. (**a** and **b**) HEK293 cells were respectively transfected with WT and mutant plasmids (T55 M, S308 T). Western blot analysis revealed that both *AP5Z1* variants decreased the level of AP-5 ζ protein. **c** ICH analysis revealed that Hela cells transfected with mutant plasmids (T55 M, S308 T) showed larger and brighter LAMP1-positive puncta as compared with cells transfected with the WT plasmid. Scale bar = 20 μm. (**d** and **e**) LAMP1 fluorescence intensity and area per cell were quantified in more than 100 cells quantified per visual field. Experiments were replicated three times. **f** Hela cells were respectively transfected with WT and mutant plasmids (T55 M, S308 T), then assessed for morphological changes by TEM. Ultrastructural analysis revealed that both *AP5Z1* variants led to the accumulation of enlarged morphologically defined endocytic structures filled with aberrant storage material, including many intraluminal vesicles. Scale bar = 500 nm. Error bars represent SEM, *p < 0.05

*AP5Z1* encode AP-5 ζ, a subunit of AP-5 complexes. AP-5 complexes play a vital role in the endosomal pathway. Functional analysis was performed in two novel variants (p.T55 M and p.S308 T) within *AP5Z1*. Western blot analysis revealed that both of *AP5Z1* variants decreased AP-5 ζ protein level (*p* < 0.0001) (Fig. 5a, b). Furthermore, Hela cells transfected with mutant plasmids showed larger and brighter LAMP1-positive puncta as compared with cells transfected with the WT plasmid (Fig. 5c). We also found that the brightness and area of LAMP1 fluorescence significantly increased in cells transfected with mutant plasmids (*p* < 0.05) (Fig. 5d, e). Transfected HeLa cells were then assessed for morphological changes by TEM. Ultrastructural analysis revealed that the accumulation of enlarged morphologically defined endocytic structures filled with aberrant storage material, including many intraluminal vesicles (Fig. 5f). Thus, the p.T55 M and p.S308 T were classified as likely pathogenic variants (PS3, PM1, PP3, PP4).

## Discussion

To date, rare studies have been conducted to investigate all causative genes for recessive HSP in the Chinese population. In this study, we performed genetic analyses in 24 unrelated AR/sporadic HSP patients by applying a combination of NGS, MLPA and Sanger sequencing. Eleven mutations including 7 novel ones in 7 known genes related to HSP were identified in 8 out of 24 unrelated cases. Among them, mutations in *ALDH18A1, AP5Z1, CAPN1,* and *ATP13A2* were first reported in the Chinese population while mutations in *SPG11*, *CYP7B1*, *and GBA2* had been reported previously [12, 13, 24].

In our study, 19 sporadic HSP and 5 AR-HSP families were included. Mutations in AR-HSP causative genes were identified in 7 sporadic and one AR-HSP families. The 7 sporadic HSP families are actually AR-HSP families after genetic testing, thus the total of AR-HSP families is 12 in the present study. Two of them carried mutations in *SPG11*. Combined with our previous study [15], the number of AR-HSP families in our cohort was 17, and the frequency of *SPG11* mutations in AR-HSP were 23.5% (4/17), which was a bit less than that in the Caucasian population (30.9%, 30/97) [9] and a recent study in the Chinese population (33.33%, 12/36) [12]. However, our study also reinforces the notion that *SPG11* is the most frequent genetic determinant in AR-HSP patients. In agreement with literature data that nonsense and frameshift mutations accounted for the majority of known *SPG11* mutations [20], two *SPG11* novel variants (p.V1979_L1980delinsX and p.F2343 fs) identified in our study were both nonsense mutations. The phenotype of *SPG11* patients usually linked with mental retardation like learning difficulties in childhood or decline in intelligence quotient (IQ) Score. In addition, thin corpus callosum and white matter abnormalities often occur in brain MRI [25, 26]. Coincided with previous reported patients [10, 27], case 1 in our study presented typical SPG11 phenotype with spastic paraplegia and mental retardation. However, case 2 in our study showed hand tremor and memory defects without signs of any other Parkinsonism, which was a rare presenting sign [28].

The nonsense mutation in *SPG5*/*CYP7B1* (p.R112X) has been reported in Chinese HSP patients from Taiwan and Fujian by independent groups [24, 29]. In the study of Taiwanese patients, this mutation was identified in one AR-HSP and four sporadic cases. Two patients presented complex phenotypes with cerebellar ataxia and three had pure phenotypes [24]. In the study of patients from Fujian, this mutation was identified in 20 HSP patients from 14 families. The frequency of *CYP7B1* mutation p.R112X in AR-HSP were 56% (14/25) in Fujian province, which was far more than that in the other area of China. Most of them presented complex form with ataxia and/or axonal peripheral neuropathy [29]. Haplotype analysis by these two groups suggested that there was a ‘founder effect’ in patients carrying p.R112X in *CYP7B1*. In our study, only one patient (1/24) carrying p.R112X was detected. Interestingly, this patient (case 3) came from Fujian province, which suggests that the ‘founder effect’ for p.R112X may exist only in the Fujian population and Taiwanese. The patient presented different phenotypes from the previously reported Chinese cases [24, 29], including spastic paraplegia with visual impairment, dysarthria and dysphagia.

*ALDH18A1* encodes P5CS, an enzyme that catalyzes the first common step of proline and ornithine biosynthesis from glutamate [30]. The phenotypes of patients with *ALDH18A1* mutations ranged from AD-HSP (SPG9A) to AR-HSP (SPG9B). Up to now, 7 SPG9A families and 5 SPG9B families with *ALDH18A1* mutations have been reported [31–34]. Mutation in *ALDH18A1* was never reported in the Chinese population. In our study, the serum P5CS activity of case 4 (SPG9B) decreased, which revealed that the novel homozygous variant (p.S242 N) identified in case 4 was pathogenic. But case 4 did not present intellectual disability which usually occurred in the previously reported patients (Table 3) [32–34].

**Table 3:** Clinical features of patients with mutations in *ALDH18A1* gene

Case No./Origin	Case 3/China^a^	FSP856/Spain [32]	SR45/Portugal [32]	HSP190/Japan [34]	HSP48/Japan [34]	NA/NA [33]
variant	p.S242 N/p.S242 N^a^	p.D715H/p.D715H	p.R128H/p.L637P	p.R441^a^/p.R665Q	p.R128H/p. L637P	p.R84Q/p.E581K
patient	1	2	4	2	2	1
AAO (mean)	12	7	< 1	< 6	32	< 1
DD (mean)	20	33	45	NA	NA	19
LL Spasticity	**+**	**+**	**+**	**+**	**+**	**+**
Pyramidal sign	**+**	**+**	**+**	**+**	**+**	**+**
Intellectual disability	**–**	**+**	**+**	**+**	**+**	**+**
Ataxia	**–**	**–**	**–**	**+**	**+**	NA
Cutis laxa	**–**	**–**	**–**	**–**	**–**	**–**

*AAO* Age at onset, *DD* Disease duration, *LL* Lower limbs, + Present, − Absent, *NA* Not available; ^a^: Present study; [32–34]: references

The *GBA2* gene was identified related to SPG46 in 2013 [35] and *GBA2* mutations were also related with progressive ataxia [36]. In 2018, a Norwegian group identified a homozygous deletion mutation in *GBA2* (p.M510Vfs*17) in families with Marinesco-Sjogren syndrome and the patients presented with ataxia, early-onset cataracts, hypotonia and muscle weakness [37]. In our study, the p.D597fs in *GBA2* identified in case 5 is the first small deletion mutation associated to HSP and the phenotype is consistent with previously reported patients [38, 39].

AP-5 complex facilitates vesicle-mediated intracellular sorting and trafficking of selected transmembrane cargo proteins and interacts with SPG11 and SPG15 [40]. Biallelic mutations in *AP5Z1* have been linked to SPG48 and the clinical features of SPG48 patients were similar to SPG11 or SPG15 patients [2, 6]. SPG48 genotypes are rare, and the onset age is variable [10]. In this study, we firstly identified SPG48/AP5Z1 mutations in the Chinese population and confirmed that they induced lysosomal dysfunction as the loss-of-function mutations of *AP5Z1* reported previously [41].

We identified *CAPN1* mutations in the Chinese population for the first time. *CAPN1* gene was identified as an AR-HSP pathogenic gene in 2016 and the patients presented complex HSP, including peripheral neuropathy, ataxia, ocular movement abnormalities, dysarthria (SPG76) [42]. The variant c.759 + 1G > A in *CAPN1* was previously identified in a family with spastic ataxia [22]. However, our patient (case 6) showed pure form, implying patients carrying *CAPN1* mutations could present either complex or pure HSP [43].

ATP13A2 is a lysosomal P5-type transport ATPase and loss-of-function mutations within *ATP13A2* cause dysfunction of lysosomal and mitochondrial. In addition, mutations in *ATP13A2* were associated with Kufor-Rakeb Syndrome (KRS), and neuronal ceroid lipofuscinosis (NCL) [44–48]. In 2017, a Germanic team first reported *ATP13A2* mutations in a patient with complex HSP (type SPG78) [49]. Here, we first reported *ATP13A2* mutations in a Chinese AR-HSP patient. Our patient (case 8) presented spastic paraplegia of lower limbs without Parkinsonism. KRS, HSP and NCL were each defined according to their most striking clinical features. KRS is a rare form of juvenile-onset atypical Parkinson disease (PARK9) associated with dementia, supranuclear gaze palsy and spasticity that has been considered to be part of the neurodegeneration with brain iron accumulation (NBIA) spectrum of disease [45]. *ATP13A2*-related NCL patients clinically present rigidity, akinesia and intellectual impairment. Post-mortem pathological examination showed abundant neuronal and glial lipofuscinosis involving the cortex, basal nuclei, cerebellum [48]. HSP (SPG78) presents complex form with cerebellar ataxia, cognitive impairment and axonal neuropathy [49]. Actually, the phenotypes of *ATP13A2*-related disease (KRS, NCL or HSP) have converged on dementia, spasticity, parkinsonism, ataxia, and peripheral neuropathy. However, why *ATP13A2* mutations associated with a spectrum of neurodegenerative disorders remains unclear.

The genetic features and clinical phenotypes of AR-HSP patients were described in our study in detail. However, no clear genotype-phenotype correlation was observed. Several reasons should be considered, the AR-HSP cases in our cohort were relatively small. In addition, the clinical phenotypes of AR/sporadic HSP were highly heterogeneous. After screened by targeted NGS, the remaining 16 AR/sporadic patients were still left without a plausible genetically diagnosis, indicating the existence of unknown genes. Besides, the targeted NGS may also loss of some valuable variants, including nucleotide repeat expansions and the variants reside outside the tested regions.

## Conclusion

In this study, we investigated 24 unrelated patients with AR/sporadic HSP by targeted NGS and functional analysis. We identified 11 mutations including 7 novel mutations in HSP related genes in 8 patients and firstly reported mutations in *ALDH18A1, AP5Z1, CAPN1* and *ATP13A2* in the Chinese population. Furthermore, the clinical phenotypes of the patients carrying mutations were described in detail. Our results expanded the genetic spectrum and clinical profiles of AR-HSP patients in the Chinese population, and further demonstrated the efficiency and reliability of targeted NGS diagnosing suspected HSP patients.

## Additional file

Additional file 1: **Table S1.** Detailed information of targeted genes included in the panel. (DOCX 42 kb)

Additional file 2: **Table S2.** Personal and medical histories of 24 patients with HSP. (DOCX 20 kb)

## Data Availability

The data supporting the conclusions of this article are available from the corresponding author upon request.

## Abbreviations

AAO: Age at onset
ACMG: American College of Medical Genetics and Genomics
AD: Autosomal dominant
AR: Autosomal recessive
EMG: Electromyogram
HSP: Hereditary spastic paraplegia
IQ: Intelligence quotient
KRS: Kufor-Rakeb Syndrome
MLPA: Multiplex ligation-dependent probe amplification
MMSE: Mini-mental state examination
MRI: Magnetic resonance imaging
NBIA: Neurodegeneration with brain iron accumulation
NCL: Neuronal ceroid lipofuscinosis
NGS: Next-generation sequencing
