# Supplementary material for: Clinical features and genetic spectrum in Chinese patients with recessive hereditary spastic paraplegia
Source: Transl Neurodegener. 2019 Jun 26;8:19. doi: 10.1186/s40035-019-0157-9 (PMC6593507; doi:10.1186/s40035-019-0157-9)
Supplement: Supplementary file 1 — Table S1. Detailed information of targeted genes included in the panel. (DOCX 42 kb) [file 40035_2019_157_MOESM1_ESM.docx]

**Additional file 1: Table S1** Detailed information of targeted genes included in the panel

| **Gene** | **Subtype** | **Inheritance** | **Locus** | **Exon** | **OMIM (Gene)** | **Refseq NM** | **Refseq NP** |
| --- | --- | --- | --- | --- | --- | --- | --- |
| *L1CAM* | SPG1 | XL | Xq28 | 29 | 308840 | NM_001278116.1 | NP_001265045.1 |
| *PLP1* | SPG2 | XL | Xq22.2 | 8 | 300401 | NM_001128834.2 | NP_001122306.1 |
| *ATL1* | SPG3A | AD/AR | 14q22.1 | 14 | 606439 | NM_001127713.1 | NP_001121185.1 |
| *SPAST* | SPG4 | AD | 2p22.3 | 17 | 604277 | NM_014946.3 | NP_055761.2 |
| *CYP7B1* | SPG5 | AR | 8q12.3 | 6 | 603711 | NM_004820.3 | NP_004811.1 |
| *NIPA1* | SPG6 | AD | 5q11.2 | 5 | 608145 | NM_144599.4 | NP_653200.2 |
| *PGN* | SPG7 | AR | 16q24.3 | 17 | 602783 | NM_003119.3 | NP_003110.1 |
| *KIAA0196* | SPG8 | AD | 8q24.13 | 29 | 610657 | NM_014846.3 | NP_055661.3 |
| *ALDH18A1* | SPG9 | AD/AR | 10q24.1 | 18 | 138250 | NM_001323412 | NP_001310341 |
| *KIF5A* | SPG10 | AD | 12q13.13 | 29 | 602821 | NM_004984.2 | NP_004975.2 |
| *SPG11* | SPG11 | AR | 15q14 | 38 | 610844 | NM_001160227.1 | NP_001153699.1 |
| *RTN2* | SPG12 | AD | 19q13.32 | 11 | 603183 | NM_005619.4 | NP_005610.1 |
| *HSPD1* | SPG13 | AD | 2q33.1 | 12 | 118190 | NM_199440.1 | NP_955472.1 |
| *ZFYVE26* | SPG15 | AR | 14q24.1 | 42 | 612012 | NM_015346.3 | NP_056161.2 |
| *BSCL2* | SPG17 | AD | 11q13 | 11 | 606158 | NM_001122955.3 | NP_001116427.1 |
| *ERLIN2* | SPG18 | AR | 8p11.2 | 12 | 611605 | NM_007175.6 | NP_009106.1 |
| *SPG20* | SPG20 | AR | 13q13.3 | 9 | 607111 | NM_001142295.1 | NP_001135767.1 |
| *ACP33* | SPG21 | AR | 15q22.31 | 9 | 608181 | NM_001127889.4 | NP_001121361.1 |
| *SLC16A2* | SPG22 | XL | Xq13.2 | 6 | 300095 | NM_006517.4 | NP_006508.2 |
| *B4GALNT1* | SPG26 | AR | 12q13.3 | 11 | 601873 | NM_001478.4 | NP_001469.1 |
| *DDHD1* | SPG28 | AR | 14q21 | 13 | 614603 | NM_001160148.1 | NP_001153620.1 |
| *KIF1A* | SPG30 | AR | 2q37.3 | 49 | 601255 | NM_001244008.1 | NP_001230937.1 |
| *REEP1* | SPG31 | AD | 2p11.2 | 7 | 609139 | NM_022912.2 | NP_075063.1 |
| *ZFYVE27* | SPG33 | AD | 10q24.2 | 13 | 610243 | NM_001002261.3 | NP_001002261.1 |
| *FA2H* | SPG35 | AR | 16q23 | 7 | 611026 | NM_024306.4 | NP_077282.3 |
| *PNPLA6* | SPG39 | AR | 19p13.2 | 34 | 603197 | NM_001166111.1 | NP_001159583.1 |
| *SLC33A1* | SPG42 | AD | 3q25.31 | 7 | 603690 | NM_001190992.1 | NP_001177921.1 |
| *C19orf12* | SPG43 | AR | 19q12 | 3 | 614297 | NM_001256047.1 | NP_001242976.1 |
| *GJC2* | SPG44 | AR | 1q42.13 | 2 | 608803 | NM_020435.3 | NP_065168.2 |
| *NT5C2* | SPG45 | AR | 10q24.32 | 18 | 600417 | NM_001134373.2 | NP_001127845.1 |
| *GBA2* | SPG46 | AR | 9p13.3 | 17 | 609471 | NM_020944.2 | NP_065995.1 |
| *AP4B1* | SPG47 | AR | 1p13.2 | 10 | 607245 | NM_001253852.2 | NP_001240781.1 |
| *AP5Z1* | SPG48 | AR | 7p22.2 | 17 | 613653 | NM_014855.2 | NP_055670.1 |
| *TECPR2* | SPG49 | AR | 14q32.31 | 20 | 615000 | NM_014844.4 | NP_055659.2 |
| *AP4M1* | SPG50 | AR | 7q22.1 | 15 | 602296 | NM_004722.3 | NP_004713.2 |
| *AP4E1* | SPG51 | AR | 15q21.2 | 21 | 607244 | NM_007347.4 | NP_031373.2 |
| *AP4S1* | SPG52 | AR | 14q12 | 6 | 607243 | NM_001128126.2 | NP_001121598.1 |
| *VPS37A* | SPG53 | AR | 8p22 | 12 | 609927 | NM_152415.2 | NP_689628.2 |
| *DDHD2* | SPG54 | AR | 8p11.23 | 18 | 615003 | NM_001164232.1 | NP_001157704.1 |
| *C12orf65* | SPG55 | AR | 12q24.31 | 3 | 613541 | NM_001194995.1 | NP_001181924.1 |
| *CYP2U1* | SPG56 | AR | 4q25 | 5 | 610670 | NM_183075.2 | NP_898898.1 |
| *TFG* | SPG57 | AR | 3q12.2 | 8 | 602498 | NM_001195478.1 | NP_001182407.1 |
| *KIF1C* | SPG58 | AD/AR | 17p13.2 | 23 | 603060 | NM_006612.5 | NP_006603.2 |
| *USP8* | SPG59 | AR | 15q21.2 | 20 | 603158 | NM_001128610.2 | NP_001122082.1 |
| *WDR48* | SPG60 | AR | 3p21.33 | 19 | 612167 | NM_001303402.1 | NP_001290331.1 |
| *ARL6IP1* | SPG61 | AR | 16p12-p11.2 | 6 | 607669 | NM_015161.2 | NP_055976.1 |
| *ERLIN1* | SPG62 | AR | 10q24.31 | 11 | 611604 | NM_001100626.1 | NP_001094096.1 |
| *AMPD2* | SPG63 | AR | 1p13.3 | 19 | 102771 | NM_001257360.1 | NP_001244289.1 |
| *ENTPD1* | SPG64 | AR | 10q24 | 10 | 601752 | NM_001164178.1 | NP_001157650.1 |
| *NT5C2* | SPG65 | AR | 10q24.32 | 18 | 600417 | NM_001134373.2 | NP_001127845.1 |
| *ARSI* | SPG66 | AR | 5q32 | 2 | 610009 | NM_001012301.2 | NP_001012301.1 |
| *PGAP1* | SPG67 | AR | 2q33.1 | 27 | 611655 | NM_024989.3 | NP_079265.2 |
| *FLRT1* | SPG68 | AR | 11q13.1 | 2 | 604806 | NM_013280.4 | NP_037412.2 |
| *RAB3GAP2* | SPG69 | AR | 1q41 | 35 | 609275 | NM_012414.3 | NP_036546.2 |
| *MARS* | SPG70 | AR | 12q13.3 | 21 | 156560 | NM_004990.3 | NP_004981.2 |
| *ZFR* | SPG71 | AR | 5p13.3 | 20 | 615635 | NM_016107.3 | NP_057191.2 |
| *REEP2* | SPG72 | AR | 5q31 | 8 | 609347 | NM_001271803.1 | NP_001258732.1 |
| *CPT1C* | SPG73 | AD | 19q13.33 | 20 | 608846 | NM_001199752 | NP_001186681 |
| *IBA57* | SPG74 | AR | 1q42.13 | 3 | 615316 | NM_001010867 | NP_001010867 |
| *MAG* | SPG75 | AR | 19q13.12 | 11 | 159460 | NM_002361 | NP_002352 |
| *CAPN1* | SPG76 | AR | 11q13.1 | 22 | 114220 | NM_001198868 | NP_001185797 |
| *FARS2* | SPG77 | AR | 6p25.1 | 7 | 611592 | NM_001318872 | NP_001305801 |
| *ATP13A2* | SPG78 | AR | 1p36.13 | 29 | 610513 | NM_022089 | NP_071372 |
| *ALS2* | IAHSP | AR | 2q33.1 | 34 | 606352 | NM_020919.3 | NP_065970.2 |
| *OPA1* | unassigned SPG | AD | 3q29 | 31 | 605290 | NM_130837.2 | NP_570850.2 |
| *OPA3* | unassigned SPG | AR | 19q13.32 | 2 | 606580 | NM_001017989.2 | NP_001017989.2 |
| *GRID2* | unassigned SPG | AD | 4q22 | 16 | 602368 | NM_001510.3 | NP_001501.2 |
| *CCT5* | unassigned SPG | AR | 5p15.2 | 11 | 610150 | NM_012073.4 | NP_001293084.1 |
| *ALDH3A2* | SLS | AR | 17p11.2 | 11 | 609523 | NM_001031806.1 | NP_001026976.1 |

AD = autosomal dominant; AR = autosomal recessive; IAHSP = infantile onset hereditary spastic paraplegia; OMIM = 0nline Mendelian Inheritance in Man;

Refseq = reference sequence; SLS = Sjögren-Larsson Syndrome; XL = X-linked.
