# Supplementary material for: Clinical features and genetic spectrum in Chinese patients with recessive hereditary spastic paraplegia
Source: Transl Neurodegener. 2019 Jun 26;8:19. doi: 10.1186/s40035-019-0157-9 (PMC6593507; doi:10.1186/s40035-019-0157-9)
Supplement: Supplementary file 2 — Table S2. Personal and medical histories of 24 patients with HSP. (DOCX 20 kb) [file 40035_2019_157_MOESM2_ESM.docx]

**Additional file 2: Table S2** Personal and medical histories of 24 patients with HSP

| **Clinical features** | **Number of patients (n = 24)** |
| --- | --- |
| Male | 17/24 (70.83%) |
| Female | 7/24 (29.17%) |
| Sporadic | 19/24 (79.16%) |
| Autosomal recessive | 5/24 (20.83%) |
| consanguinity | 9/24 (37.50%) |
| Pure HSP | 7/24 (29.17%) |
| Complex HSP | 17/24 (70.83%) |
| Age at onset (years) | 20 (1-58) |
| Duration of disease (years) | 11 (1-54) |
| **Pyramidal signs** |  |
| LL hyperreflexia | 22/24 (91.67%) |
| UL hyperreflexia | 13/24 (54.17%) |
| Babinski | 14/24 (58.33%) |
| Hoffmann sign | 7/24 (29.17%) |
| Ankle clonus | 14/24 (58.33%) |
| **Additional signs** |  |
| LL weakness | 9/24 (37.50%) |
| Pes cavus | 2/24 (8.33%) |
| Neuropathy | 7/24 (29.17%) |
| Cerebellar signs | 6/24 (25.00%) |
| Mental retardation | 5/24 (20.83%) |
| Tremor | 3/24 (12.50%) |
| Visual impairment | 1/24 (4.17%) |

UL: upper limbs; LL: lower limbs
